# Healthcare Disparities and the Impact on Mortality in Incarcerated Patients

**DOI:** 10.7759/cureus.71660

**Published:** 2024-10-16

**Authors:** Abdul Muqtadir, Jai Kumar, Wayne Diah, Sayed Husain

**Affiliations:** 1 Internal Medicine, Shadan Institute of Medical Sciences, Hyderabad, IND; 2 Internal Medicine, Dow University of Health Sciences, Karachi, PAK; 3 Nephrology, Infigo Dialysis, LLC, Sanford, USA

**Keywords:** chronic kidney disease (ckd), community hospital, correctional, dialysis, healthcare disparities, incarcerated patients, mortality

## Abstract

This article addresses the healthcare disparities and potential impact on mortality among incarcerated patients with chronic kidney disease (CKD). Incarcerated individuals are more likely to suffer from acute and chronic health conditions and have inconsistent access to healthcare services. Demographic trends, behavioral health trends, and lack of insurance coverage are major contributing factors to healthcare disparities among incarcerated individuals. This article highlights the need for a new model of care, which includes clinical programs focusing on the transition period from incarceration to the community and linking individuals to post-incarceration healthcare utilizing community health workers and clinicians to establish rapport with individuals prior to their release. Addressing healthcare disparities and providing adequate healthcare to incarcerated individuals is crucial because a conviction should not deprive an individual of basic human rights, including the right to healthcare.

## Introduction and background

Chronic kidney disease (CKD) is defined as abnormal kidney function for longer than three months with a glomerular filtration rate (GFR) of less than 60 ml/minute/1.73 m^2^. This condition affects about 15% of the adult population in the United States, that is one in seven adults [1]. Incidence, prevalence, and progression of CKD also vary by ethnicity and social determinants of health. CKD is more common in non-Hispanic Black adults (20%) than in non-Hispanic Asian adults (14%) or non-Hispanic White adults (12%) [2]. Disadvantaged populations are disproportionally affected by unequal access to healthcare; additionally, incentives to promote early intervention in advanced disease beyond provisional care are still developing [3].

Most people with CKD risk factors are unaware of the progression of their disease since renal symptoms often appear later in life. Several CKD risk factors including hypertension, diabetes, cardiovascular problems, autoimmune disease, and stroke cause an increased risk of premature mortality [4]. Renal failure is three times more prevalent in Black individuals compared to White individuals; however, the diagnosis requires specialized lab techniques [5]. These inequalities are due to various factors including a higher prevalence of risk factors including obesity, tobacco use, poverty, and limited access to primary and specialist care [6].

The United States has an incarcerated population of approximately 1.9 million which is mainly composed of ethnic and racial minorities, in addition to individuals with a history of substance abuse, smoking, and alcohol [7]. These groups have a high prevalence rate of CKD. Demographic details, effectiveness, and outcome of self-administered continuous ambulatory peritoneal dialysis (CAPD) in correctional facilities were analyzed revealing complications such as fluid leaks, peritonitis, and cardiac events [8]. However, these efforts were not studied, and no published data was provided.

The availability of dialysis services in correctional institutions varied by state. Full dialysis services are provided on-site in 24 states, 10 states report a combination of on-site and off-site services, and 10 states have off-site services [9]. Obesity is linked with several long-term health conditions associated with chronic kidney disease. Obesity disproportionately impacts Black inmates more than their Caucasian counterparts, and incarcerated females compared to males [10].

This review highlights kidney disease areas that reveal the largely unknown consequences of incarceration. It summarizes published literature on incarcerated individuals with CKD and highlights possible approaches for lowering the severity of renal disease in this overly-impacted population. It is of utmost importance to realize the significance of the healthcare disparity between incarcerated and non-incarcerated people and to address them regardless because a conviction does not deprive a person of basic human rights, one of which is the right to healthcare.

## Review

Methodology

Literature was reviewed from Scopus, Google Scholar, and PubMed, including articles published up to August 2023 and excluding language limitations. Various keywords were utilized and MeSH (Medical Subject Headings) such as 'correctional’, ‘incarcerated’, ‘community hospital’, ‘dialysis’, ‘ESRD’, ‘chronic kidney disease’, and ‘mortality’. Article abstracts were assessed for relevant information consistent with the objectives and aims of this review. Complete texts of screened literature were carefully reviewed for abstraction and inclusion of relevant data into this literature review.

Results

Demographic Trends

Domestic shifts obscure the significant incarceration differences in local communities, cities, and across states. A limited number of communities are unequally impacted by mass incarceration as a United States criminal justice policy. Poverty, joblessness, family instability, and urban, African American racial segregation are closely connected to mass incarceration. The correlation between concentrated poverty and later incarceration is significant; however, the link between concentrated poverty and the level of crime is equivalent to the disadvantage and imprisonment. These findings suggest that communities that disproportionately produce prisoners are also more likely to disproportionately draw prisoners back upon release. This could assist in clarifying the substantial amount of consistency and the core predicament in over-incarcerated communities [11].

A criminal record hinders a person’s attained socioeconomic status, and preexisting disadvantage compounds the limited socioeconomic opportunities available to released offenders. However, few studies have thoroughly examined the extent of involvement in the correctional system needed to negatively impact socioeconomic opportunities [12]. From the data of the National Longitudinal Study of Adolescent to Adult Health, the authors examined how prior criminal justice experiences shape social mobility and how ascribed socioeconomic status mitigates the connection between involvement in the United States correctional system and achieved socioeconomic status [12]. Following release from incarceration, the consequences of a criminal record emerge when evaluating an individual’s social and economic accomplishments. A history of criminal convictions impedes employment opportunities and seeking further education; however, developing a relationship with employers may reduce the harmful effects of a criminal history.

Individuals in correctional facilities tend to suffer from past behavioral health risk factors. Substance use disorder is associated with a greater incidence of significant health problems, including chronic kidney disease. From 2007-2009, 58% of individuals in state prisons and 63% of people with jail sentences fulfilled the requirements of substance abuse or drug dependence when compared to the general population aged 18 or older, which was 5% [13]. More than 33% of incarcerated people suffer from chronic health conditions, as per numerous major studies focusing on health within the incarcerated population. When chronic medical conditions percentages are broken down by correctional facility type, 36% of federally incarcerated people, 43% of individuals imprisoned in state institutions, and 39% incarcerated locally suffer from chronic medical conditions [14].

New Model of Care Analytic Strategy

Researchers have found that many factors affecting incarceration level also affect population health, and thus it is important to account for the endogenous nature of incarceration rates when examining the effects of incarceration rates on health. In examining the relationship between incarceration rates and population health at the county level, researchers found that the predicted incarceration rate is positively associated with worse health outcomes, and higher predicted incarceration rates are associated with higher levels of mortality and morbidity. They also found that incarceration expenditures have significantly expanded; however, its budget share only substantially impacts Public Assistance but no other programs. Although public health outcomes are linked to total welfare spending, the crowding-out of expenditure on the correctional system should be proportionally moderated, and there is a weak positive association between total corrections spending and Public Assistance. The incarceration rate as a function of total correctional system spending and sociodemographic factors was determined and then assessed for population health outcomes [15].

Although there are challenges, a renal transplant program for prisoners can be successfully sustained resulting in better clinical outcomes and cost benefits when compared to hemodialysis [16]. All prisoners are entitled to proper medical care under the United States Constitution; however, transplantation is not ethically questionable. Approval of transplants for inmates is determined by the prison administration on an individual basis. Despite this, the ethical committee of the Organ Procurement and Transplantation Network recommends that "one's position as a prisoner should not exclude them from receiving a transplant." Nevertheless, the Acquisition and Transplantation of Organs Network admits that more non-medical aspects may affect a patient's eligibility for a transplant and gives the transplant recipient specific listing decision programs [16]. Consequently, the software decides what to list. Without consistent standards, officials are reluctant to assess detainees and waitlist them.

Discussion

Factors Contributing to Healthcare Disparities Among Incarcerated Patients

Limited access to healthcare services is a major factor contributing to healthcare disparities in incarcerated people. Incarcerated individuals often have inadequate access to medical facilities and healthcare professionals within correctional facilities [17]. This causes a significant barrier to equitable care. Overcrowding, resource limitations, and logistical challenges also contribute to delayed or denied medical attention.

Inadequate screening and diagnosis contribute to healthcare disparities in the incarcerated population. Many inmates enter correctional facilities with preexisting health conditions that often go undiagnosed or undertreated [14]. Correctional institutions often lack proper medical screenings and comprehensive health assessments on entry [18]. This further exacerbates existing health disparities in inmates.

Mental health neglect imparts healthcare disparities in incarcerated people. A disproportionately high number of incarcerated individuals struggle with mental health issues, yet mental healthcare services are often insufficient within the prison system. This neglect contributes to increased suicide rates and exacerbates underlying mental health conditions [19].

Racial and socioeconomic disparities contribute to healthcare disparities among incarcerated patients. Incarcerated populations are disproportionately composed of individuals from minority and economically disadvantaged backgrounds. These groups often face heightened healthcare disparities even before incarceration, leading to compounding health challenges while imprisoned [20].

Inadequate chronic disease management also contributes to healthcare disparities in incarcerated individuals [18]. Proper management of chronic diseases, such as diabetes and hypertension, is often hindered by subpar medical infrastructure within correctional facilities. This lack of management increases the risk of mortality [21].

The confluence of these factors results in higher mortality rates among incarcerated individuals compared to the general population. Studies have consistently shown elevated mortality rates due to preventable and treatable conditions such as infectious diseases, cardiovascular diseases, and substance use disorders [22].

Addressing Healthcare Disparities

Comprehensive health screenings would be a significant measure in addressing healthcare disparities. Implementing thorough health screenings upon entry can help identify and address preexisting health conditions, ensuring timely interventions [23]. Improving mental health services by prioritizing mental health services, including assessments, counseling, and treatment, can reduce suicide rates and improve overall mental well-being among inmates [24].

Equitable resource allocation with adequate funding and resource allocation to correctional healthcare can help bridge the gap between incarcerated patients' healthcare needs and the services provided [25]. Additionally, collaboration with community providers can help to address healthcare disparities. Strengthening partnerships between correctional facilities and community healthcare providers can ensure post-release continuity of care, reducing the likelihood of relapse or exacerbation of health conditions [26]. Education and training can also alleviate healthcare disparities. Providing training to correctional facility staff on healthcare protocols and cultural competence can enhance the quality of care provided to incarcerated patients [27].

A chance to enhance health and meet the medical requirements of underserved groups exists with the integration of healthcare in correctional institutions and the public healthcare system [28]. There is insufficient data to conclusively say that handing over control of health services in correctional facilities to the Ministry of Health will improve service delivery, continuity of care, patient safety, and population health outcomes [29]. In their capacities as health managers, leaders, advocates, and healthcare professionals are crucial to the transformation of care for this population.

The Health and Retirement Study (HRS) analyzed chronic disease burden changes in 8,872 non-Hispanic Black, non-Hispanic White, and Hispanic participants. The study revealed that non-Hispanic White people and non-Hispanic Black individuals start with a higher degree of chronic illness burden and experience multimorbidity at a younger age [30]. Conversely, when compared to non-Hispanic White individuals, Hispanic people develop chronic illness more rapidly [31]. These findings have important implications for bolstering efforts to prevent primary and secondary chronic diseases among Hispanic Americans and non-Hispanic Black individuals to reduce the negative health effects of multimorbidity.

Behavioral Health Trends

Incarcerated individuals tend to also suffer from past behavioral health risk factors including drug and alcohol abuse, which are linked to a greater prevalence of significant health problems which include chronic kidney disease. From 2007-2009, 58% of people in state prisons and 63% of people convicted to jail met the requirements of substance dependency or abuse compared to 5% in the overall population aged 18 years or more [13]. Numerous major studies focusing on health within the incarcerated population have found that more than 33% of incarcerated people suffer from chronic health conditions, which includes 36% of those federally imprisoned, 43% of individuals in state prisons, and 39% of people in local correctional facilities. The prevalence of a diabetes diagnosis was 11% in federal inmates, 10% in state inmates, and 8% in locally incarcerated individuals, in contrast with 7% in the overall United States population [14].

Transplant and Dialysis

There is limited peer-reviewed data on incarcerated patients receiving dialysis and kidney transplants. Additionally, prisoners are precluded from many transplantation programs due to logistics and transportation difficulties which prevent obtaining laboratory results at short notice [16].

Post-Prison Medical Coverage

Two weeks following release from prison, the risk of death is greatest and considerably higher in comparison to the overall population. All-cause mortality is high among recently released prisoners, with drug overdose as the leading cause after cyclic vomiting syndrome (CVS), which is linked to kidney function [32]. Lack of insurance coverage along with the stigma associated with incarceration, challenges in locating a healthcare provider, problems maneuvering the medical system, and social and economic obstacles that occur following release from incarceration [33-35].

Prospective New Model of Care

One treatment paradigm concentrates on the interim time from imprisonment to release into the community. Recently released inmates are linked to medical services following incarceration utilizing medical staff and health professionals in the community to develop rapport before their release. Medical professionals then assist with post-incarceration appointments and aid with maneuvering through their complicated social and economic situation, with an emphasis on safeguarding the health of individuals recently released from incarceration [26].

## Conclusions

The incarcerated community in the United States is disproportionately affected by CKD, and their access to healthcare services is inconsistent. This is likely due to demographic and behavioral health factors that make this population more susceptible to chronic diseases. Access to healthcare services is inconsistent and data on medical specialty care, including dialysis facilities, are rather limited. Furthermore, it is noted that the cost of providing dialysis to the incarcerated is relatively higher than the general population due to security reasons. The risk of mortality is greatest in the first two weeks following release from incarceration, with drug overdose being the most common cause of death. Lack of insurance coverage, stigmatization associated with imprisonment, challenges in locating a healthcare provider, problems maneuvering the medical system, and social and economic barriers create post-release obstacles for individuals.

However, new models of care concentrating on the transitional period from imprisonment to release into the community can help to connect these individuals with healthcare following incarceration utilizing medical staff and health professionals in the community to develop rapport before their release. Health professionals can assist with post-incarceration appointments and aid with maneuvering through their complicated social and economic situations, which cause post-release challenges for an individual. It was noted that the decision to provide kidney transplants to prisoners is a complex issue that involves weighing medical, logistical, and ethical considerations. It is essential to address the underlying healthcare disparities between incarcerated individuals and the general population and better realize the significance of the basic human right to healthcare regardless of conviction.
